# Severe Allergic Reactions to Food in Norway: A Ten Year Survey of Cases Reported to the Food Allergy Register

**DOI:** 10.3390/ijerph8083144

**Published:** 2011-07-26

**Authors:** Ellen Namork, Christiane K. Fæste, Berit A. Stensby, Eliann Egaas, Martinus Løvik

**Affiliations:** 1 Division of Environmental Medicine, Norwegian Institute of Public Health, P.O. Box 4404, Nydalen, Oslo NO-0403, Norway; E-Mails: berit.a.stensby@fhi.no (B.A.S.); martinus.lovik@fhi.no (M.L.); 2 National Veterinary Institute, Oslo N-0106, Norway; E-Mails: christiane.faste@vetinst.no (C.K.F.); eliann.egaas@vetinst.no (E.E.); 3 Norwegian University of Science and Technology, Trondheim NO-7491, Norway

**Keywords:** severe reactions to food, IgE-mediated reactions to food, food allergy register, food allergens

## Abstract

The Norwegian Food Allergy Register was established at the Norwegian Institute of Public Health in 2000. The purpose of the register is to gain information about severe allergic reactions to food in Norway and to survey food products in relation to allergen labelling and contamination. Cases are reported on a voluntary basis by first line doctors, and submitted together with a serum sample for specific IgE analysis. The register has received a total of 877 reports from 1 July, 2000 to 31 December, 2010. Two age groups, small children and young adults are over-represented, and the overall gender distribution is 40:60 males-females. The legumes lupine and fenugreek have been identified as two “new” allergens in processed foods and cases of contamination and faults in production of processed foods have been revealed. The highest frequency of food specific IgE is to hazelnuts and peanuts, with a marked increase in reactions to hazelnuts during the last three years. The Food Allergy Register has improved our knowledge about causes and severity of food allergic reactions in Norway. The results show the usefulness of population based national food allergy registers in providing information for health authorities and to secure safe food for individuals with food allergies.

## 1. Introduction

Western countries have experienced an increase in the prevalence of asthma and allergies in the last decades. Admissions for food allergy have also increased with some evidence of a rise in prevalence of food allergy in children [1,2]. Although the prevalence of perceived food allergy is generally much higher than can be verified with the established diagnostic methods [3], food allergy with potentially life-threatening anaphylactic reactions still constitutes a significant public health problem. There is, however, a lack of information about the incidence of serious food allergic reactions, risk situations, incriminating foods and causative allergens, treatment given and patient follow-up. In most nationwide surveys, data are based on telephone interviews and questionnaires giving a picture at the time of the study, often for a specific allergen and a selected group of the population. Some national and regional registers do exist in Europe but the structure and mode of operation vary.

In Europe, the first register was started in Sweden in 1993 when physicians were asked to report life-threatening and fatal allergic reactions caused by food as they occurred. A total of 215 allergic reactions were registered up to 2008 [4]. In the year 2000, a Finnish register of severe allergic reactions was established at the Skin and Allergy Hospital of the Helsinki Central Hospital. A one-page questionnaire form is available on the Internet and data on 530 cases, independently of the causative agent, was recently published [5].

The French National Allergy Vigilance Network was created by CICBAA (Circle of Clinical and Biological Investigations in Food Allergy) in 2001, based on reports from private and hospital allergologists [6]. The network has 456 members throughout France and other French speaking countries. A recent register of anaphylaxis (Network for Online Registration of Anaphylaxis, NORA) comprising Germany, and the German-speaking parts of the countries Austria and Switzerland, was started in 2005. The data are collected through an online questionnaire and up to January 2009, 1,163 anaphylactic reactions have been reported [7]. An Italian surveillance system for regional anaphylaxis, the Veneto Region Registry for Anaphylaxis (VRRA), was established in North-Eastern Italy in 2006. The system is a web-based real-time interactive system monitoring a population of 4.5 million.

Of the existing registers, the Finnish register is the closest in mode of operation to the Norwegian Food Allergy Register presented below. The Norwegian register, however, is unique in that all first-line doctors submitting a report are involved, including emergency wards and out-patient clinics, after attending to the patient.

### 1.1. The Food Allergy Register in Norway

The Norwegian National Reporting System and Register of Severe Allergic Reactions to Food (the Food Allergy Register), was established 1 July, 2000 at the Norwegian Institute of Public Health in collaboration with the Norwegian Food Safety Authority (NFSA) and the National Veterinary Institute (NVI). An earlier report describing the structure and purpose of the register has been published [8]. In short; the Food Allergy Register is government funded and constitutes a nationwide, voluntary system for reporting of severe allergic reactions to food. Severe reactions are defined as reactions causing medical help to be sought within 24 hours after intake of the offending food. Reactions should be reported without waiting for further diagnostic verification. Cases are reported on a one-page paper form, with the possibility to submit supplementary information by physicians, covering family doctors, specialists, emergency wards and relevant hospital departments. For each case, a consent form must be signed by the patient. The forms and serum samples are submitted to the Food Allergy Register for registration and specific IgE analysis and when relevant, food samples are sent to NVI for allergen analysis. Printed information material and forms are mailed to physicians with relevant practices approximately every other year, and are also available on the internet.

The main purpose of the Food Allergy Register is to gain information about severe allergic reactions to food in Norway, and to generally increase the knowledge about food allergy in the society. It is also important to survey that the presence of food allergens in processed foods is in compliance with food labelling directives in order to increase food safety for individuals suffering from food allergy. Results that require official action are reported to the NFSA.

## 2. Materials and Methods

The reports contain information about the patient such as gender, age, known allergies, former allergic reactions, medications if any, and a short case history including the suspected or incriminating food, the circumstances under which the reaction took place, symptoms and the treatment given.

### 2.1. ImmunoCap™

The Food Allergy Register is routinely testing sera against a standard panel of 12 food allergens and two pollen allergens using the ImmunoCap™ 100 automated system (Phadia, Uppsala, Sweden). At present, the standard panel comprises the following food allergens; milk, wheat, egg, pea, soy, hazelnut, peanut, fenugreek, shrimp, celery, cod, and salmon. The sera are also routinely tested against pollen allergens from birch and timothy grass. Additional serum specific IgE antibodies to food and pollen allergens are analysed as judged relevant for the individual case. The standard panel gives valuable statistical information for these key allergens and helps in the diagnosis. Strength of IgE-specific binding is recorded in kU/L and as defined classes from 1 to 6 (0.37 ≥ 100 kU/L). Classes 1–6 includes all specimens with detectable IgE, classes 2–6 (0.7 ≥ 100 kU/L) includes sera with IgE levels of possible clinical significance, and the classes 3–6 comprise sera with high specific IgE levels (>3.5–>100 kU/L).

### 2.2. Allergen Analysis of Food

As found appropriate in relation to the case history and serology, food extracts made from the suspected foods are analysed qualitatively and quantitatively by ELISA and Western blotting using polyclonal or monoclonal antibodies, or by proteomic analysis using gel electrophoresis and mass spectrometry. Reference foods are used for comparison and verification. Information on the presence of allergens, including undeclared, “hidden” or novel allergens is used to support the case diagnosis and is also communicated to the NFSA. If necessary, NFSA will contact the producer of the food product.

### 2.3. Feedback to the Reporting Physician

Physicians submitting case reports receive the results of the specific serum IgE analysis and food allergen analysis when performed, together with an evaluation of the case. Each case is discussed with focus on relevant food allergens and allergies, cross-reactions, risk foods and symptoms. A standard passage informs that a positive serological result does not warrant a diagnosis of an allergy unless the corresponding clinical symptoms are present, and that food should not be eliminated from the diet without a medical reason. The physician is also informed that the Food Allergy Register should be considered as a supplement to the diagnostic work performed locally. The laboratory analysis, the discussion and advice for each case are a complementary service to the reporting physician and the patient.

### 2.4. Statistics

Frequency analyses and plots of the collected data are made using PASW^®^ Statistics 17.0 and SigmaPlot 11.0.

## 3. Results and Discussion

### 3.1. Frequency of Reporting

**Figure 1.**
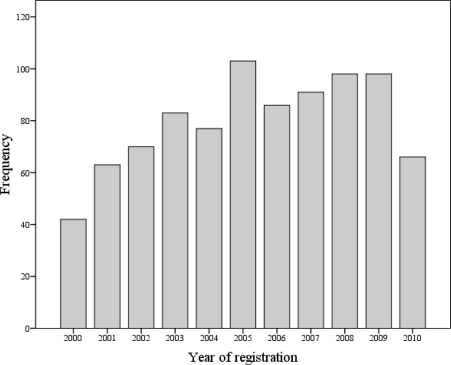
Reported cases of severe reactions to food in Norway registered from 1 July, 2000 to 31 December, 2010 (The number for 2000 contains reports for 6 months only).

The Food Allergy Register has received 70–90 reports per year (Figure 1) adding up to a total of 877 reports from 1 July, 2000 to 31 December, 2010.

The variation in number of annual reports may reflect the level of awareness amongst the physicians and patients about the existence of the Food Allergy Register due to the distributed information material and information in media. In 2005, a comprehensive information campaign was performed by sending data sheets and report forms to all relevant physicians (approximately 6,500), clinics and hospitals in Norway which resulted in a noticeable increase of registered cases that year (Figure 1). Hence, the lower number of reports received in 2010 indicates the need for another information campaign. The same physicians in the same clinics and hospitals are noted to repeatedly report cases indicating that they find the Food Allergy Register useful. This, on the other hand, points to the fact that there exist a nationwide underreporting from physicians in other practices, clinics and hospitals.

Differences in the geographical distribution of the registered cases are also noted. The report frequencies of the three biggest cities in Norway are 55 reports/100,000 inhabitants in the capital city Oslo, lowest in Bergen, which is the second largest city, with only 6.5 reports/100,000 inhabitants and 68 reports/100,000 inhabitants in Trondheim which has the lowest number of inhabitants. The report frequency may, therefore, reflect differences in geography and lifestyle, diet and the availability of facilities for allergy diagnosis and treatment in that area rather than number of inhabitants. The commitment of the respective doctors to contribute to a central register, as well as differences in awareness of the patients themselves may also be important factors. The discrepancy concerning regional report frequencies supports the assumption of a considerable underreporting making it difficult to give an estimate of the true number of cases of severe allergic reactions in Norway. An increase in yearly reports may therefore reflect an increase in the frequency of reporting and not a rise in incidences of severe food allergic reactions.

### 3.2. Gender Distribution

The results show an age-dependent gender distribution. From early adulthood there is a constant ratio of 40:60 male-female, in contrast to small children showing the opposite ratio of 60:40 boys: girls. Similar differences in gender are recognised in several questionnaire-based studies in other countries [5,9] and are suggested to reflect a higher incident of self-diagnosed allergy for females than males. In the present register, however, the records are based on severe allergic reactions reported by the attending physicians indicating the necessity of medical care. The higher incidence of food allergy in women is, therefore, likely to have a physiological cause. In an Australian study, females were reported to outnumber males in both acute allergic reactions and anaphylaxis [10], supporting this assumption. The observed age-related gender difference is similar to that reported for asthma, hay fever and atopic disease, suggesting that puberty and the influence of sex hormones may have an important impact on the prevalence of atopic diseases in general [11].

### 3.3. Age Distribution

**Figure 2.**
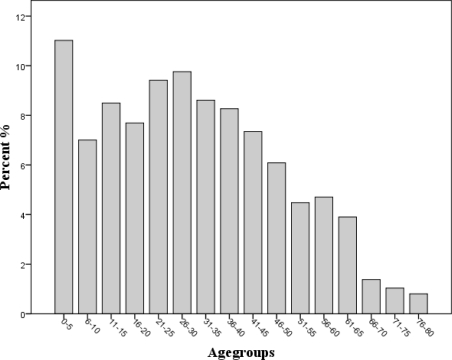
The age distribution shows two risk groups; one from 0 to 5 and one from 21 to 35 years of age.

The age distribution among the 877 reports received has also been constant over the years. Two risk groups are apparent; one group comprising the smallest children aged zero to five years and one broader group of young adults aged between 21 and 35 (Figure 2). It is well known that childhood food allergy most often manifests itself in the first two years of life and falls progressively until late childhood. An increased risk for fatal food allergic reactions among young adults is also reported in other studies [12]. It is reasonable to assume that a possible explanation for the young adults comprising a risk group is a more exploratory behaviour. They represent an age group that frequently goes to parties, often eats out and has little control of the ingredients in the food they are eating. Furthermore, risks may be taken under the influence of alcohol and food may be eaten that previously has caused a reaction. Additionally, sensitization to aeroallergen begins at young age, increases during childhood and reaches a peek in young adults for tree pollen sensitisation [13], representing a possible risk of accompanying cross-sensitisation to plant foods. The possibility of a parallel increase in primary food allergies cannot be ruled out.

### 3.4. Analysis of Blood Samples

**Figure 3.**
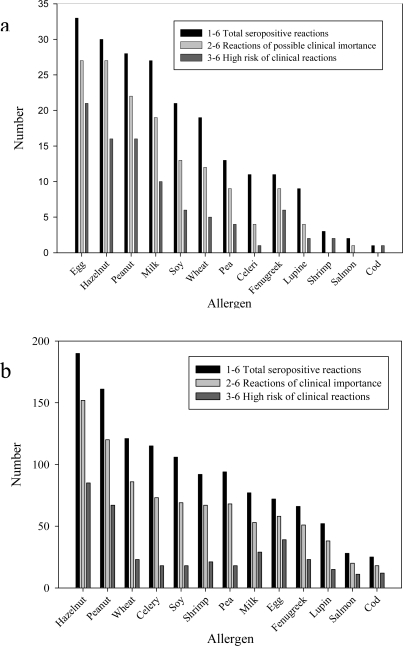
(**a**) Egg, hazelnut, peanut and milk are the food allergens with the highest sensitisation rate in the small children’s group (0–5 year). (**b**) Hazelnut and peanut are the food allergens with the highest sensitisation rate overall as measured by specific IgE in the patient sera.

A full blood or serum sample for specific IgE analyses follows the reports in about 90% of the cases. Although quantification of specific IgE in predicting clinical reactivity has been found useful for egg, milk, peanuts and fish in specific settings [14], the limited predictive value of sero-positive reactions in relationship to clinical allergy and the limitations in methodology must be remembered. In the present register, the frequencies of specific IgE to various allergens are found to differ between the two risk groups. As found in most other reports [15–17], egg, milk, hazelnut and peanut are the allergens most often causing IgE sensitisation in infants and small children (Figure 3a). However, the foods with the highest frequency of specific IgE overall, are hazelnut, peanut, wheat and celery (Figure 3b). Figure 3, also indicates that IgE-mediated reactions to fish are rare. Norwegians have long traditions for a high consume of fish and the lack of sensitisation may be due to early tolerance development, or to underreporting by especially the coastal cities and communities, that have a low reporting frequency in general.

**Figure 4.**
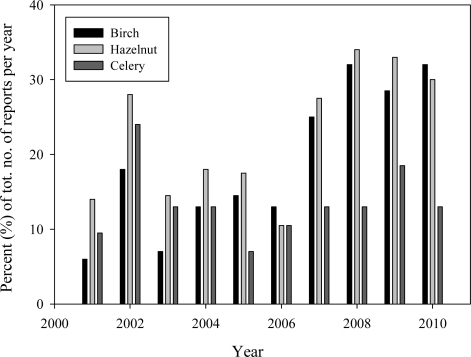
Year to year variation in specific IgE to birch, hazelnut, and celery from 2001 to 2010 (Note the marked increase in birch and hazelnut from 2007 to 2010).

In Scandinavia, sensitisation to both hazelnuts and celery often develops due to a primary birch allergy. By looking at the year to year variations in specific IgE reactions for all the single allergens in the standard panel, an increase is seen for hazelnut and birch, from 2001 to 2010, and to a lesser degree for celery, (Figure 4). This observation suggests a rise in allergy to birch pollen with following cross-sensitisation to plant foods such as hazelnuts and celery, indicating an overall increase in incidences of cross-reactions among the cases reported. The number of reports with positive specific IgE to birch, hazelnut and celery is given in percent of the total number of reports per year, to compensate for the difference in the yearly total number. Linear Regression analysis confirmed a significant increase during the last 10 years of specific IgE to birch (95% CI: 1.53–4.13, *p* = 0.001) and hazelnuts (95% CI: −0.011–3.62, *p* = 0.05), but not to celery. Component based analysis, using the recombinant hazelnut allergen Cor a1, and celery allergen Api g1, both homologues of the birch pollen allergen Bet v1, support that these reactions are cross-sensitisations due to primary allergy to birch pollen [18]. The observed trend is in line with the overall high incidence of pollen related food allergies reported in Europe and especially in Scandinavia [19,20]. The increase in sensitisation to pollen may possibly be explained by global warming and longer pollen seasons [21,22].

### 3.5. Food Samples

Samples of the incriminating or suspected food may be useful to confirm the cause of a reaction but are forwarded in only 8–10% of the reported cases. Analysis of food has, however, in some cases revealed the presence of allergens stated specifically not to be present, or the accidental presence of food allergens due to errors in the food production, or contamination of food in the production line. Examples are milk found in sausage-meat declared not to contain milk causing anaphylactic reaction in a patient allergic to milk, egg ovalbumine in egg-free marzipan, fishcake contaminated by shellfish [23] and sweet cake containing cocoa contaminated with hazelnuts causing anaphylaxis in a patient allergic to hazelnut.

After the introduction of lupine flour to bakery and pastry products in Norway, the Food Allergy Register received a number of reports of peanut allergic patients experiencing serious reactions caused by lupine supplementing wheat flour in bakery products [24], or by lupine contamination in the production-lines. Labelling of lupine has now become mandatory partly due to register-based observations. Fenugreek is another legume recently discovered to be an allergen of importance in Norway. In 2005 to 2006, the Food Allergy Register received a number of reports about peanut allergic patients experiencing reactions to spicy sauces and Indian dishes. The reactions were found to be caused by the aromatic seeds of fenugreek, a common ingredient in curry and other mixed spices [25]. These are examples of new and so called “hidden” allergens introduced in processed foods in Norway during the last decade.

The high prevalence of specific IgE to peanuts and hazelnuts in the Norwegian population resembles the findings in other Nordic countries [4,5] and in Great Britain and France [26,27], as well as in the United States [2,3], pointing out peanuts and tree-nuts as the leading cause of severe allergic reactions and with increasing prevalence. The increase and severity of peanut allergy may have serious consequences with regard to cross-reactions to other legumes such as lupine and fenugreek, since clinically relevant cross-reactions between peanuts and these two legumes often seem to occur [25,28,29].

### 3.6. Onset of Reaction

The one-page questionnaire form to be filled in by the physicians, requests information on the time between intakes of the suspected food to the onset of symptoms. In 70% of the cases where this information is given, 61% of the reactions are reported to occur within 30 minutes. A relationship between time to onset of symptoms and positive serological IgE-specific reactions was also observed. Later onsets of symptoms may often, but not always, be caused by other mechanisms than IgE-mediated allergy.

### 3.7. Treatment Given

70% of the case-reports include information on the treatment given and show that 46.3% are treated with adrenaline alone or in combination with steroids and antihistamines. Another 32% are given injections of steroids alone or in combination with antihistamines, suggesting that most of these reactions are also considered severe by the physicians.

### 3.8. Experiences after Ten Years of Operation

Some reporting doctors find the case evaluations useful and report regularly, others report less often, and some are not aware of the register’s existence. Hence, the results from the register are not quantitative. In spite of the variation in reporting throughout the country, the register appears to be representative since both the gender and age distribution have been constant over the years and are in agreement with other studies in Europe. The discovery of lupine and fenugreek in processed foods and information given to the media of the risk of severe cross-reactions in peanut allergic individuals after intake of these allergens substantially diminished the reports, proving the usefulness of the register in preventing serious reactions to foods. A trend is also registered of an increase in birch pollen allergies during the last few years, with following cross-reactions to plant foods such as to hazelnut and celery.

## 4. Conclusions

The Food Allergy Register has given valuable information about severe allergic reactions to food in Norway. The Food Allergy Register has revealed food safety problems in relation to allergy such as contamination and faults in production of processed foods. Furthermore, the discovery of the “new” legume allergens has contributed to the mandatory labelling of lupine. For future food allergy risk assessment and food labelling policies, better information is needed on food allergen properties, prevalence of allergic reactivity in the population and exposure factors. Population-based national registers of severe food allergic reactions should therefore be established to protect the consumers at risk of potentially life-threatening allergic reactions.

Analyses of existing regional population-based studies and registers regarding the type and quality of the collected data are currently being used to develop a plan for a pan-European register [30]. Such a register of allergic reactions will improve the way allergies, in particular food allergies, as a common cause of anaphylaxis can be prevented and managed in Europe.
